# Evaluation of a pilot of a community virtual triage for breast symptoms outside of usual primary or secondary care pathways

**DOI:** 10.1308/rcsann.2023.0094

**Published:** 2024-02-26

**Authors:** S Laws, K Spiller, C Glew

**Affiliations:** ^1^ Hampshire Hospitals NHS Trust, UK; ^2^ Wessex Rapid Investigation Service, UK; ^3^ Winchester Rural North and East Primary Care Network, UK

**Keywords:** Mastalgia, Breast services, Cancer referrals

## Abstract

Both primary and secondary care services in the NHS have been overwhelmed with an increase in referrals on the suspected cancer pathways. The years 2020/2021 saw 551,770 symptomatic breast referrals made in England alone. The Wessex Rapid investigations service in conjunction with the local district general hospital and primary care networks instigated a virtual triage for new breast symptoms. Over the course of a year, 664 people were assessed by either telephone or video using specially trained nurses. Appointments were given within 1–2 working days. The service was highly valued by patients and general practitioners. We were unable to confirm a reduction in referral to secondary care as the evaluation occurred during a postpandemic peak in referrals. We found that 10% of patients with new breast symptoms can safely self-manage. This percentage varied with the experience of the triage clinician. A specialist community face-to-face service could reduce further the need for full secondary care evaluation. Better integration and use of information technology systems would improve the service. The rapid responsiveness and length of consultations is valued by patients. Representation with the same symptoms was rare. This pathway utilises staff outside of the usual primary and secondary care providers and thus reduces the pressure on stretched systems.

## Introduction

Breast symptomatic referral pathways have been under unprecedented pressure, with units across England struggling to meet targets. The years 2020/2021 saw 551,770 symptomatic breast referrals made.^1^ Alongside this, general practitioner (GP) practices have been struggling to give timely appointments. About 20% of referrals to secondary care are for breast pain alone and could have been managed without referral.^2^ Reducing the number of referrals to secondary care will improve the capacity for rapid cancer diagnosis. Education and self-management for patients with breast pain can reduce both anxiety and need for clinical evaluation.

In conjunction with local primary care networks (PCNs) and a secondary care breast unit, the Rapid Investigation Service (RIS) developed a virtual-based triage system. The aims were to facilitate rapid referral of patients with red flag symptoms, to triage patients for GP review and to provide advice, guidance and education for self-management with appropriate safety nets.

Across the UK, breast units have developed multiple pathways to ease the demand for breast secondary care evaluation. These pathways are now being evaluated concurrently using the ASPIRE collaborative audit.^3^ To our knowledge, our pathway is the only one to date that has developed the service with primary care and uses staff outwith the usual service providers, thus increasing overall capacity.

## Methods

The breast self-referral pilot service formed part of the Wessex Cancer Alliance (WCA) response to the national Rapid Diagnostic Centre (RDC) Programme. The RDC programme formed part of work in place nationally and regionally to deliver faster cancer diagnosis and to improve patient experience. This has now been subsumed into the broader national Faster Diagnosis Framework.

After obtaining the appropriate governance permissions, the triage project was managed by the RIS, a large district general hospital (DGH) and the PCNs. The triage pathway and operational procedure documents went through the standard governance processes at both the host trust for the RIS and the DGH, which included care group, divisional and cancer board approval. Information governance approval was also affirmed.

Two PCNs were included: a group of six rural practices and one single large city practice, with a total registered population of approximately 88,000. Virtual triage (video call or telephone) was performed by specially trained nurses at RIS. Over the pilot, a number of Band 6 and band 7 nurses as well as a GP (for emergency cover) have provided triage. The nurses have experience in the management of telephone consultations (as part of the RIS team) and have the requisite communications skills. The nurses were also trained in the management of breast symptoms by observation in virtual and in face-to-face clinics. Most nurses completed training within a few weeks. A weekly debriefing session was available with the consultant lead and rapid email case discussions. The aim was to offer appointments every weekday where possible, among other duties, with the equivalent of two clinics per week dedicated to this service. Triage appointments were 30–45min (dependent on the experience of the clinician and needs of the patient) and gave time for health promotion and discussion of family history anxiety compared with a standard GP appointment of 10min.

People over 30 years old, excluding cisgender males, with new breast symptoms were signposted to the virtual triage service without any previous practice review. This follows the criteria for symptomatic suspected cancer referrals. A virtual appointment was given mostly within one working day. A clinical proforma designed by the primary and secondary care leads was followed and people were allocated to either a) a direct referral to secondary care, b) an appointment booked with their GP or c) were given advice and written guidance on self-management of breast pain. A follow-up appointment was arranged at an interval of two to six weeks as a safety net for the latter group and a further appointment could be made at this stage if required.

An independent group, Wessex Voices, interviewed a random sample of people from the first 3 months using a semi-structured telephone conversation to feedback on their experience of the service. All patients using the service were asked for their explicit consent to take part in a structured interview and for their details to be passed on to the external company to carry this out. This was recorded in their patient record; 31 gave consent to be interviewed. GP practices were asked to evaluate their satisfaction with the service via an emailed questionnaire; 13 out of 17 GPs responded. Secondary care clinician feedback was limited as triage pilot referral was not flagged at one-stop clinics.

We were unable to definitively evaluate whether the number of referrals to secondary care was affected by the service because of a postpandemic 150% rise in referrals over the region. Participating practices comprised approximately 15% of the catchment of the DGH.

In the DGH, the clinic notes were reviewed by experienced clinicians to determine whether people could potentially have been managed in primary care. It is acknowledged that this is an estimate and reasons for referral are complex. Patients deemed not to automatically need secondary care investigations included patients whose symptoms had resolved within two weeks (the two-week wait referral pro-forma includes a section where referral is encouraged only after a period of re-examination at a different stage of the menstrual cycle or if pain is unresolved at six weeks after simple measures) and those who did not require any secondary care investigation.

## Results

Of the 31 people interviewed by Wessex Voices, 29 were satisfied with the RIS breast service; 2 had issues at the GP practice. Most (*n*=29) said they would recommend it to others. The speed with which they were seen provided people with reassurance and allayed their anxieties.

Of the primary care review, 100% of responding GPs were conscious that the service had decreased their workload. Some GPs received comments or feedback about the service from patients, and all cited these as having been positive; 100% of GPs responded that they would be keen for the service to continue in their area.

Only two patients in the first six months selected randomly (every sixth patient from each GP practice) for audit had re-presented in primary care; both in the context of generalised anxiety unrelated to the breast.

**Figure 1:**
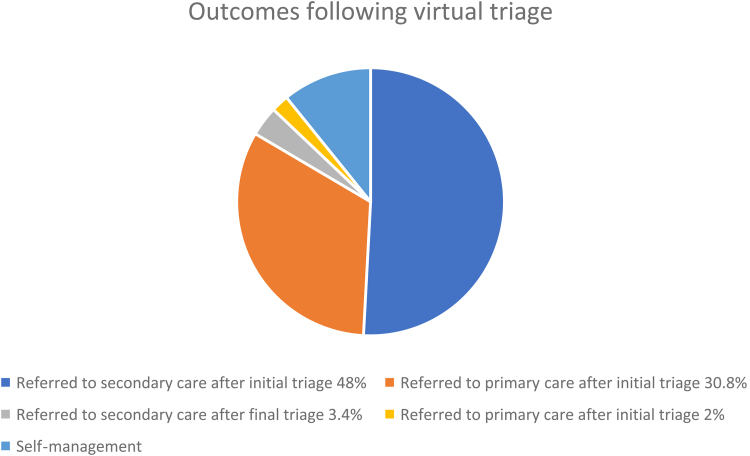
Outcomes of those triaged by RIS. Most people (78%) have final decisions regarding further assessment at first consultation. Self-management patients had a safety net follow-up call where onward referral can be made as required. RIS = rapid investigation service.

In the first year of the project, 664 people were assessed by the triage service, of whom 60% presented with a lump, 31% with pain and 9% with other symptoms. Of those reporting pain only, 27% (62/228) were given advice and guidance and self-managed. The rate of self-management appeared to vary with the RIS clinician experience. Overall, 10% were discharged with advice and guidance. To date no cancers have been detected in those triaged to self-management (Figure 1).

A total of 94 people were referred back to the GP for further assessment of self-reported symptoms, most in the context of uncertain self examination or potential infection. In total, 229 patients were assessed in primary care, of which 96 (41%) subsequently had one-stop secondary care appointments. Five patients were diagnosed with cancer after GP review and subsequent secondary care assessment.

During the first year, 325 people were referred to secondary care for fast-track assessment and seen in the NHS at the participating DGH (others chose to be seen by alternative providers). These were referred directly from the RIS to secondary care without a further GP appointment. Thus, most people waited no more than 16 days from reporting a symptom to secondary care assessment. The majority of these were lumps but this also included pain with possible additional symptoms and skin changes. Some local GP practices are able to accommodate women with breast symptoms for primary care review on the day of presentation whereas some offer appointments only up to three weeks later. The virtual triage process thus either takes an equivalent amount of time to refer to secondary care or is significantly swifter.

A total of 19 people assessed in secondary care were diagnosed with breast cancer, which is a 5.8% conversion rate.

Following audit, it was considered that 109/325 (33%) patients could potentially have been managed in primary care. These included patients whose symptoms had resolved, who had skin lesions or a normal clinical examination.

**Figure 2:**
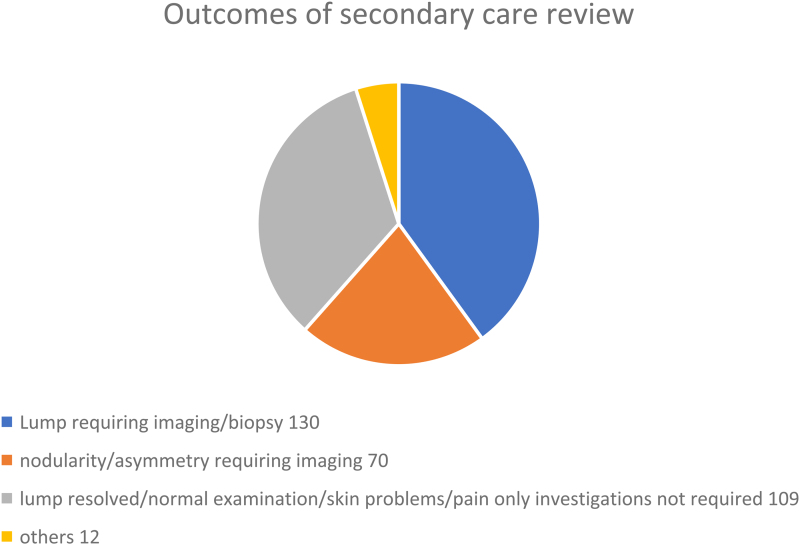
Final problem classification after secondary care assessment. Each category may contain a variety of diagnoses both benign and malignant (325 assessments).

Forty-nine patients seen in the secondary care one-stop service had a clinical examination only and no further investigations were requested (<40 years of age and with no clinical signs). A further 60 women had mammography before meeting with the clinician as per the operational policy of the one-stop service (>40 years old with no mammography in preceding six months). As these women had normal clinical examination and no red flag symptoms at review, mammography was not indicated and could be considered “opportunistic screening”. All 109 appointments took place in clinics with full radiology backup (Figure 2).

## Discussion

Breast symptoms represent a significant proportion of fast-track suspected cancer referrals to secondary care. Both primary and secondary care are struggling to cope with the inexorable rise in people with breast symptoms. Anecdotal reports (UK wide) suggest that the majority of women presenting with breast symptoms are referred directly to the breast one-stop service from primary care without recommended actions such as review at a different stage of the menstrual cycle or attempts at self-management. People with breast pain as the only symptom are unlikely to have cancer, with a 0.4% incidence compared with 0.8% through screening.^4^ This is reflected in the most recent National Institute for Health and Care Excellence (NICE) guidance (2021) where breast pain only is not a criteria for two-week wait referral.^5^ Patients with breast symptoms do need to be assessed promptly because of high levels of associated anxiety. Data suggest this often correlates with a perceived high family risk of breast cancer.^6^

This project aimed to improve time from reporting of symptoms to full evaluation, to reduce anxiety and to support self-management. The virtual triage allowed appointment time to allay anxiety. Patient consultations in the virtual triage system are stored in the GP record.

This project used additional staff outside of usual primary and secondary care pathways, increasing overall capacity. No staff were taken away from primary or secondary care breast teams to support this service. This is a significant benefit to pressurised systems. The triage nurses are also trained to provide triage for rapid access to other cancer services.

There are a number of limitations to this pathway, not least that some patients choose to have face-to-face contact only. Some patients are unable to participate due to disabilities, but the standard primary care pathways remain available. Only patients with new symptoms were eligible for this service. Patients with a past history of breast cancer either have direct access to the secondary care team or go through the GP for a more detailed consultation.

It can be difficult to determine over the telephone whether a lesion is on the skin of the breast or in the breast parenchyma. Patients are often uncertain as to whether they actually have a lump or not. These patients required face-to-face assessments.

Any symptoms that suggested an acute infection were referred back for rapid assessment by the GP rather than waiting for a two-week wait referral in case emergency treatment was required.

We have had to recruit additional staff to support this service and this has required both training costs and supervision costs from the senior team members. We are currently evaluating whether this service is cost-effective and should be commissioned. Initially only 15% of the local DGH catchment was involved in the pilot. This has now been extended to 40%, which should make the service more cost-effective.

Managerial aspects of the project were reviewed three-monthly and with weekly clinical support. Navigation of the information technology (IT) systems used by different organisations was challenging. Improvement of back office functions has allowed us to reduce the amount of administration support for this service, for example, by users inputting demographic data online.

A face-to-face community clinic has commenced. This focuses on symptom management, evaluation of family risk plus lifestyle changes to reduce breast cancer. We have now recruited two (trainee) advanced nurse practitioners (Band 7) who provide the specialist telephone triage as well as the face-to-face consultations. They have also been trained to be independent in the secondary care one-stop clinics. This has improved the capacity in both primary and secondary care.

To evaluate both the telephone triage, focusing on self-management and the face-to-face community clinic, we are taking part in the Association of Breast Surgery (ABS) ASPIRE project.

## Conclusions

The breast virtual triage service allows rapid onward referral to secondary care for those with appropriate symptoms. The conversion rate of referral to cancer (5.8%) is equivalent to rates in England for suspected cancer referrals (5.7%).

There is a considerable reduction in the number of people that need to be reviewed by the GP for breast symptoms.

Symptoms, where breast pain only, can be self-managed with appropriate advice and guidance. Longer appointments allow for better reassurance. This appears to reduce re-presentations.

Additional refinement is underway to attempt to reduce the number of people referred on to secondary care that could have potentially been managed without a full one-stop service, for example, those uncertain about self-examination.

Both patients and GPs have evaluated this service highly and we are now in the process of evaluating its costs and cost effectiveness with a view to commissioning the service.
